# Autoantibodies and vitamin D in leprosy patients in the Brazilian Amazon

**DOI:** 10.1186/s12879-026-12578-2

**Published:** 2026-01-24

**Authors:** Glaucielen Gomes da Silva, Tinara Leila de Souza Aarão, Lucas Corrêa Modesto, Luis Arthur Moreira Ferreira, Pablo Rodrigues Nunes de Souza, Rafael Malcher Meira Rocha, Luiz Fábio Magno Falcão, Juarez Antônio Simões Quaresma

**Affiliations:** 1 Postgraduate Program in Parasitic Biology of the Amazon, Center for Biological and Health Sciences, State University of Pará, Campus. II Travessa Perebebuí, CEP: 66.087-670, Marco, Belém, Pará 2623 Brazil; 2 Department of Pathology, State University of Pará, Belém, Pará Brazil; 3 Center for Biological and Health Sciences, State University of Pará, Campus II. Travessa Perebebuí, CEP: 66.087-670, Marco, Belém, Pará 2623 Brazil; 4 Center for Biological and Health Sciences, Students at State University of Pará, Campus II. Address: Travessa Perebebuí, Marco, Belém, 2623, CEP: 66.087-670 Pará Brazil; 5 Department of Pathology, Paulista School of Medicine, Federal University of São Paulo, São Paulo, Brazil; 6 University of the State of Pará, Campus VIII,Avenida Hiléia, s/n, Amapá, Marabá, Pará, CEP: 68.502-100, Brazil

**Keywords:** Leprosy, Autoimmunity, Vitamin D

## Abstract

**Introduction:**

Leprosy can vary in clinical forms, resulting in dermatoneurological disease with physical disabilities and leprosy reactions. This reaction involves complex immune system activity that can be influenced by vitamin D activity. The objective of this study was to evaluate the presence of autoantibodies in leprosy patients in Marabá, associating it with sociodemographic aspects and vitamin D levels.

**Materials and methods:**

Cross-sectional study with leprosy patients treated at a Family Health Unit. Surveys of autoantibodies, vitamin D, and sociodemographic aspects were conducted.

**Results:**

Most patients were male (63.4%), aged equal or over 15 years old (80.5%), brown (68.3%), incomplete elementary education (41.5%) and earning between US$203.88 and US$407.76. The presence of autoantibodies was identified, with the most prevalent being anti-β2-GPI IgM and ANA (AC-2, AC-4, and AC-20), both in 14.6% of participants, with statistical significance in the positivity of anti-β2-GPI IgM in multibacillary patients. The average vitamin D level was 29.3 ng/mL, with 43.8 ng/mL for tuberculoid, 28.5 ng/mL for borderline, and 24.9 ng/mL for lepromatous.

**Conclusion:**

Our study demonstrated the presence of autoantibodies in leprosy patients in Marabá, more frequently in the lepromatous clinical form, and low levels of vitamin D in reactional states.

**Graphical Abstract:**

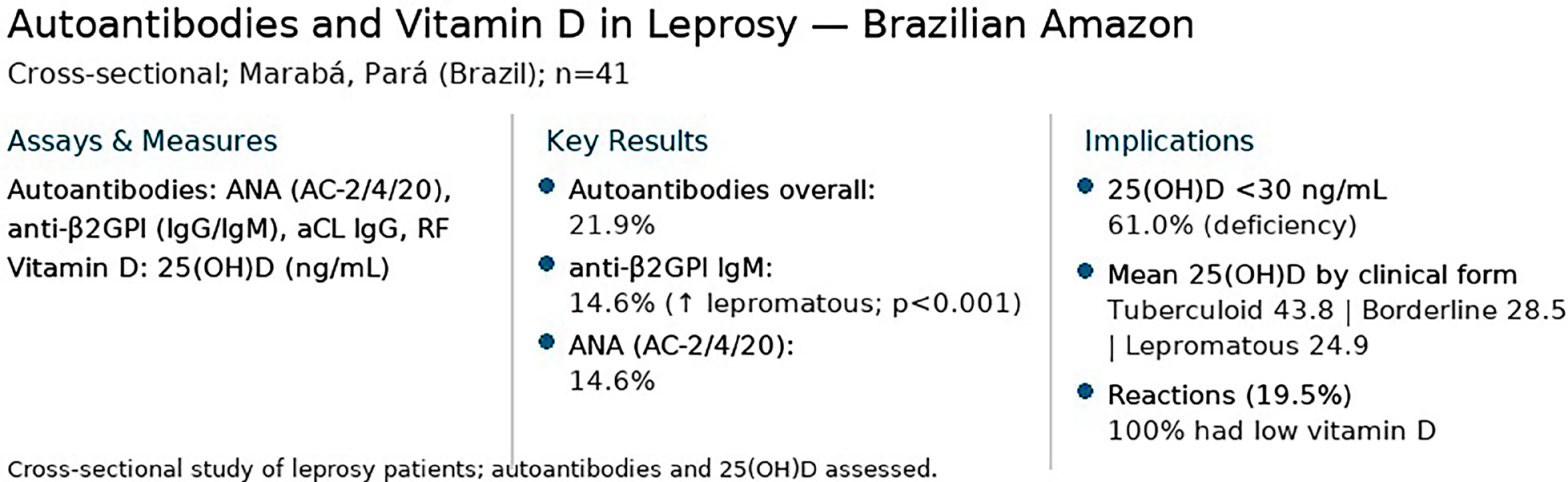

## Introduction

Leprosy, caused by Mycobacterium leprae and Mycobacterium lepromatosis, is one of the oldest human diseases and is characterized mainly by skin lesions and peripheral nerve damage [1]. Its development depends on a complex interaction between host and pathogen, with main risk factors including close and prolonged exposure to infected individuals, genetic traits influencing immune efficiency, and contact with wild animals such as armadillos [2–4]. The disease presents a clinical spectrum determined by characteristics of the infectious agent along with the host’s genetic and immunological profile.

Multiple studies have explored immune responses to M. leprae and M. lepromatosis, revealing significant modulation of innate and adaptive mechanisms [5]. In the innate response, macrophages act as primary target and effector cells, eliminating bacilli through phagocytosis [5]. Adaptive immunity is also altered throughout infection, involving both cellular and humoral pathways regulated by T helper 1 (Th1) and T helper 2 (Th2) lymphocytes, respectively [6].

In Brazil, leprosy diagnosis is based on dermatoneurological evaluation, supported by bacilloscopy and histopathology, and classified according to the Madrid system into paucibacillary (indeterminate, tuberculoid) and multibacillary (borderline, lepromatous) forms [7]. Tuberculoid and lepromatous represent the stable mild and severe poles, while indeterminate and borderline are considered unstable. Immunological reactions may occur before, during, or after treatment, classified as type 1 or type 2, the latter potentially progressing to Lúcio’s phenomenon, a rare condition characterized by intense bacillary load, vasculitis, and thrombosis [7, 8].

The type 1 (R1) reaction, known as reverse reaction, can occur in paucibacillary or multibacillary patients and presents as mild fever and infiltration of existing lesions that become more erythematous and inflamed [9, 10]. The type 2 (R2) reaction usually presents as erythema nodosum lepromatosum (ENL), affecting patients with multibacillary lepromatous, presents with systemic manifestations such as fever and the presentation of inflamed and painful lesions, which can lead to joint involvement [1, 4, 8, 9]. Both are acute reactions mediated by activities of the immune system, such as inflammation, innate response cells, activity of T lymphocytes, B lymphocytes, *natural killer* cells (NK), production of pro-inflammatory cytokines and antibodies [4, 5]. These reactions present a clinical condition that can easily be confused with autoimmune rheumatic diseases.

Some conditions may hinder leprosy diagnosis, including dermatophytosis, pityriasis versicolor, vitiligo [11], rheumatoid arthritis, and systemic lupus erythematosus (SLE) [12], as they share immunopathological features that can lead to diagnostic confusion and require careful differential evaluation [11]. Autoantibody positivity has also been documented in leprosy: RF, ANA, and anti-aCL were reported in a histoid leprosy [13], and studies in Amazonian populations identified RF, ANA, ANCA, and antiphospholipid antibodies such as anti-aCL and anti-β2GPI [14, 15]. ANA and anti-aCL have also been observed in children and adolescents with leprosy [16].

Some mechanisms associate infectious diseases with the autoimmune response, including molecular mimicry, presentation of new, modified or cryptic antigens, superantigens and activation of bystander autoreactive lymphocytes [5, 17].

Studies indicate that, beyond pathogen–host interactions, vitamin D plays an important role in both innate and adaptive immunity, influencing susceptibility to infectious and autoimmune diseases. In innate defense, its active form (1,25OHD) induces antimicrobial peptides such as cathelicidins and β-defensins, which act directly against bacterial cell walls [18], and also participates in antiviral and antifungal responses [19]. Vitamin D has been shown to stimulate macrophage autophagy [20], regulating inflammation and tissue repair, while its receptor (VDR), expressed in T and B lymphocytes, modulates cytokine release and immune profiles [21]. However, this response depends on VDR functionality, which may be impaired by polymorphisms. Variants such as FokI [22, 23], TaqI [23–25], ApaI [22, 25] and BsmI in haplotypes [26] have been associated with increased susceptibility to infection.

The relationship between vitamin D and leprosy has been the subject of studies and research. It is understood that vitamin D activity can generate immunoregulation, reducing injuries and reactions caused by the immunological response against *M. leprae*. This process involves genetic inheritance, as well as direct biological response and environmental factors [27], since low doses of vitamin D and antimicrobial peptides, as well as low expression of VDR have been reported in patients with leprosy [28, 29], in addition to hypovitaminosis D during leprosy reaction conditions [30], although serious doses of vitamin D have not shown a significant interaction with the clinical forms of leprosy [23].

As previously stated, the clinical evolution of leprosy can vary in clinical forms, which can result in neurological disease that will result in disabilities, in addition to acute immunological reactions, leprosy reactions, which affect individuals and can generate limitations in their development, as well as in work activity. and economical. This development involves activity of the immune system, and, therefore, this study aimed to identify the presence of autoantibodies in the blood of individuals with leprosy, associating it with clinical forms, leprosy reactions, vitamin D dosage and epidemiological characteristics.

## Materials and methods

This study consisted of a cross-sectional analysis and was based on the application of a form to sociodemographic informations, on sex, age (< 15 years and ≥ 15 years), education and income, origin, residence, housing conditions and basic sanitation, clinical form (Madrid classification), existence of leprosy reactions and treatment to evaluate the epidemiology of leprosy in Marabá, in addition to collecting blood samples for screening autoantibodies (ANA, RF and antiphospholipid antibodies) and measuring vitamin D in patients diagnosed with leprosy at a main unit Family Health Public Unit in Marabá to diagnose, treat and monitor patients with leprosy and is located in a leprosy cluster area with a high number of cases [31].

The study included all patients diagnosed with leprosy by the public health service of Marabá, before, during or after (up to 6 months) treatment, excluding those patients with a previous diagnosis of an autoimmune disease. As it is a study with human beings, the work was submitted to the Research Ethics Committee of the Center for Tropical Medicine (NMT)/Federal University of Pará (UFPA) and the Research Ethics Committee of the State University of Pará, Campus VIII, and was approved under protocol numbers 3,966,220 and 3,851,670, respectively.

Autoantibody screening, ANA HEp-2, was performed on samples from all participants by indirect immunofluorescenc/HEp-2 (American type culture collection CCL-23) with serum diluted from 1:80. Tests for anti-phospholipid antibodies (anti-aCL IgG and anti-β2GPI IgG and IgM) were carried out using the enzyme immunoassay technique and turbidimetry to test for rheumatoid factor (RF). The measurement of 25-hydroxyvitamin D was performed by chemiluminescence. Since leprosy is a chronic inflammatory disease with actions that alter the functionality of the vitamin D, the reference value used for research participants was > 30.0 ng/mL.

Electronic files were created for storage and preparation of tables with the help of Microsoft^®^ Excel^®^ 2016 software and subsequent data analysis and graph creation using Biostat version 5.3. A descriptive and exploratory analysis of the main sociodemographic variables and clinical forms of the participants was performed, followed by an evaluation of data related to autoantibody and vitamin D research. Categorical data were analyzed using descriptive statistical measures (mean, median, percentages) and subjected to the chi-square goodness-of-fit test. Non-categorical data, such as vitamin D dosage, were subjected to the Shapiro-Wilk normality test, whose results varied according to the clinical form: Dimorphic *p* = 0.0097, Indeterminate/Tuberculoid *p* = 0.9803, and Lepromatous *p* = 0.2311. Therefore, for the association between clinical forms, the non-parametric two-tailed Mann-Whitney test was performed for the association of two independent groups, and the Kruskal-Wallis test for the association of three independent groups. For the analysis of autoantibody data, Fisher’s exact test was used, taking into account the small sample size. The tests were applied with a 95% confidence interval, and analyses with a p-value < 0.05 were considered statistically significant.

## Results

### Sociodemographic, clinical and treatment data

The study included the participation of 41 participants diagnosed with leprosy and treated Public Family Health Unit in Marabá, Pará between October 2020 and August 2021, the majority (63.4% *n* = 26) being male. Of these, six (14.6%) were classified as paucibacillary, one (2.4%) in the indeterminate form and five (12.2%) in the tuberculoid form, and 35 (85.4%) multibacillary participants were classified as borderline (*n* = 26 63.4%) and lepromatous (*n* = 9 21.9%), therefore there was a higher prevalence of multibacillary cases in the borderline clinical form according to the Madrid classification. The samples collected were from 12 (29.3%) patients before treatment, 18 (43.9%) during treatment and 11 (26.8%) with treatment completed within 6 months. No statistical difference was seen between the sexes (*p* = 0.0858), the average age was 35 years, with a predominance of individuals with over 15 years old (*p* < 0.0001), brown (*p* < 0.0001). Sociodemographic data are shown in Table 1.

It is possible to state that 34 (82.9%) do not have a Family Health Unit in their neighborhood and that 18 (43.9%) stated that they do not have a visit from the community health agent. Only one (2.4%) participant stated that he lived in a rural area. The most common form of diagnosis was by spontaneous demand (*n* = 36 87.8% *p* < 0.0001) and the period from onset of symptoms to effective diagnosis lasted more than 24 months (*n* = 16 39.0% *p* = 0.0972). Even though smear microscopy on intradermal scrapings is an important method for diagnosing leprosy, 20 (48.8%) of the participants did not perform it and among those who performed the test, 13 (31.7%) were negative and eight (19.5%) were positive with a bacillary index ranging from 2 to 6 crosses (2 + to 6+), the latter presented the lepromatous clinical form. All participants stated that they were strictly following the treatment schedule without rejection or resistance.

**Table 1:** Sociodemographic, economic and housing data of research participants diagnosed with leprosy and treated at a family health unit in Marabá, Pará

Variables	*n*	%	*p*-value	Variables	*n*	%	*p*-value
**SOCIODEMOGRAPHIC PROFILE**				**HOUSING PROFILE**			
**Sex**			0.0858	**Number of rooms**			
Male	26	63.4		1 room	1	2.4	0.0002*
Female	15	36.6		2 rooms	9	22	
**Age**				3 rooms	5	12.2	
< 15 years old	8	19.5	< 0.0001*	4 rooms	19	46.3	
≥ 15 years old	33	80.5		More than 4 rooms	7	17.1	
**Color**			< 0.0001*	**Number of residents**			
Yellow	1	2.4		Up to 3 residents	20	48.8	0.8759
White	3	7.3		More than 3 residents	21	51.2	
Indigenous	1	2.4		**Supply water**			
Brown	28	68.3		Wide mouth well	33	80.5	< 0.0001*
Black	8	19.5		Semi-artesian well	2	4.9	
**Education**			0.0025*	Artesian well	6	14.6	
Complete primary education	11	26.8		**Water treatment for consumption**			
Incomplete elementary education	17	41.5		Filtered	12	29.3	0.1951
Complete high school	6	14.6		Mineral	10	24.4	
Incomplete high school	3	7.3		None	19	46.4	
None	4	9.8					
**ECONOMIC PROFILE**							
**Income**							
Less than US$203.88	9	22	0.0089*				
US$203.88 and US$407.76	19	46.3					
More than US$407.76	9	22					
No income	4	9.8					

US$203.88 is approximately the minimum wage in Brazil in 2021. *statistically significant *p* < 0.05 in Test Chi-square of goodness-of-fit – X²

Until the moment of diagnosis, nine (21.9%) participants reported having undergone home treatment such as cinnamon, boldo and lemon teas, in addition to drinking bottles and using antifungal ointments. It is worth mentioning that six (14.6%) participants had been diagnosed or suspected of other diseases before leprosy, including heart failure, white cloth, rheumatism and even chickenpox. Regarding rheumatic and muscular symptoms, 19 (46.3%) reported feeling joint pain and 16 (39.0%) weakness or muscle pain.

Regarding prevention and early diagnosis, 13 (31.7%) did not have a BCG vaccination scar (Bacillus *Calmette-Guérin*), two (4.9%) were under 15 years old, another 19 (46.3%) had only one scar and nine (21.9%) had two scars. Previous interaction with individuals diagnosed with leprosy was mentioned by 30 (73.2%) participants, 27 (65.9%) reported living with multibacillary and two (7.3%) paucibacillary. Furthermore, 18 (43.9%) reported living at home, with 22 (53.6%) contacting relatives. It is worth mentioning that of the 18 participants who stated that they lived at home, only nine (21.9%) reported having carried out a contact test at the time of their relative’s diagnosis, and of the total samples (*n* = 41), the contact test was reported by 12 (29.3%) participants.

### Presence of autoantibodies

The presence of autoantibodies was identified in nine (21.9%) participants, some samples were positive for more than one type of autoantibody. Therefore, the prevalence of antibodies was calculated based on the number of autoantibodies found, the result was 37.5% (*n* = 6) for ANA, 6.2% (*n* = 1) for IgG anti-cardiolipin antibody, 12, 6% (*n* = 2) IgM antibody anti -β2-glycoprotein-I, 37.5% (*n* = 6) for the anti-β2-glycoprotein-I IgG antibody and 6.2% (*n* = 1) for rheumatoid factor. The values presented in Table 2 refer to the total number of participants.

We found that four (9.8%) participants were positive, with a titer of 1/80, for the AC-4 fluorescence pattern, which corresponds to the nuclear fine speckled pattern, and this pattern is related to the presence of anti-SSA/Ro autoantibodies and anti-SSB/La. The presence of the AC-2 pattern, which corresponds to the nuclear dense fine speckled pattern, at titer 1/320, was also verified in one participant (2.4%), this pattern is demonstrated in the presence of anti-DFS70 antibodies. Only one (2.4%) participant showed ANA positivity with a mixed fluorescence pattern, AC-4/AC-20, both with a titer of 1/320. The AC-20 pattern corresponds to the cytoplasmic fine speckled pattern that can be visualized in the presence of anti-Jo-1 antibodies.

**Table 2:** Autoantibody survey of leprosy in research participants at a family health unity in Marabá, Pará

Autoantibody	*n*	%
ANA HEp-2	6	14.6
Reagent (AC-2)	1	2.4
Reagent (AC-4)	4	9.8
Reagent (AC-4/AC-20)	1	2.4
Non-Reactive	35	85.4
**Anti-cardiolipin IgG (aCL)**		
Positive (> 15 GPL)	1	2.4
Negative (< 15 GPL)	40	97.6
**Anti -β2 glycoprotein IgG (U/mL)**		
Positive (> 5.0 U/mL)	2	4.9
Negative (< 5.0 U/mL)	39	95.1
**Anti -β2 glycoprotein IgM (U/mL)**		
Positive (> 5.0 U/mL)	6	14.6
Negative (< 5.0 U/mL)	35	85.4
**Rheumatoid Factor**		
Positive (> 14.0 IU/mL)	1	2.4
Negative (< 14.0 IU/mL)	40	97.6

ANA: anti-cell autoantibodies

Table 3 presents the relationship between positivity in autoantibody tests and the patient’s clinical form, classified as indeterminate, tuberculoid, borderline, or lepromatous. Fisher’s exact test was applied, and statistical significance was determined for the presence of ANA and anti-β2GPI IgM autoantibodies in lepromatous patients (*p* = 0.0148 and *p* = 0.0000, respectively) when compared to the other groups.

**Table 3:** Relationship between the clinical form and positivity for autoantibodies in participants diagnosed with leprosy and treated in Marabá, Pará

Autoantibody	Clinical Form							
	I (*n* = 1)	T (*n* = 5)			B (*n* = 26)		L (*n* = 9)	
**ANA HEp-2**								
Positive	0	0		2				4
Negative	1	5		24				5
*p-value*				0.1683				0.0148*
**Anti-cardiolipin IgG**								
Positive	0	0		0				1
Negative	1	5		26				8
*p-value*				0.3659				0.2195
**Anti-β2 glycoprotein IgG**								
Positive	0	0		1				1
Negative	1	5		25				8
*p-value*				1.0000				0.3951
**Anti-β2 glycoprotein IgM**								
Positive	0	0		0				6
Negative	1	5		26				3
*p-value*				0.0011*				0.0000*
**Rheumatoid Factor**								
Positive	0	0		0				1
Negative	1	5		26				8
*p-value*				0.3659				0.2195

*statistically significant *p* < 0,05 (Fisher’s exact test). ANA: anti-cell autoantibodies; I: indeterminate; T: tuberculoid; B: borderline; L: lepromatous

The prevalence of leprosy reactions was 19.5% (*n* = 8), with five (12.2%) presenting R1 and three (7.3%) R2. Among the eight participants who were in a reactive state, four were positive for autoantibodies, which characterizes a prevalence of 50.0% of autoantibodies among the reactive participants, with some being positive for more than one autoantibody. It is worth noting that all participants with R2 were positive for autoantibodies. The small sample size made it impossible to perform statistical tests.

### Vitamin D dosage

**Fig. 1:**
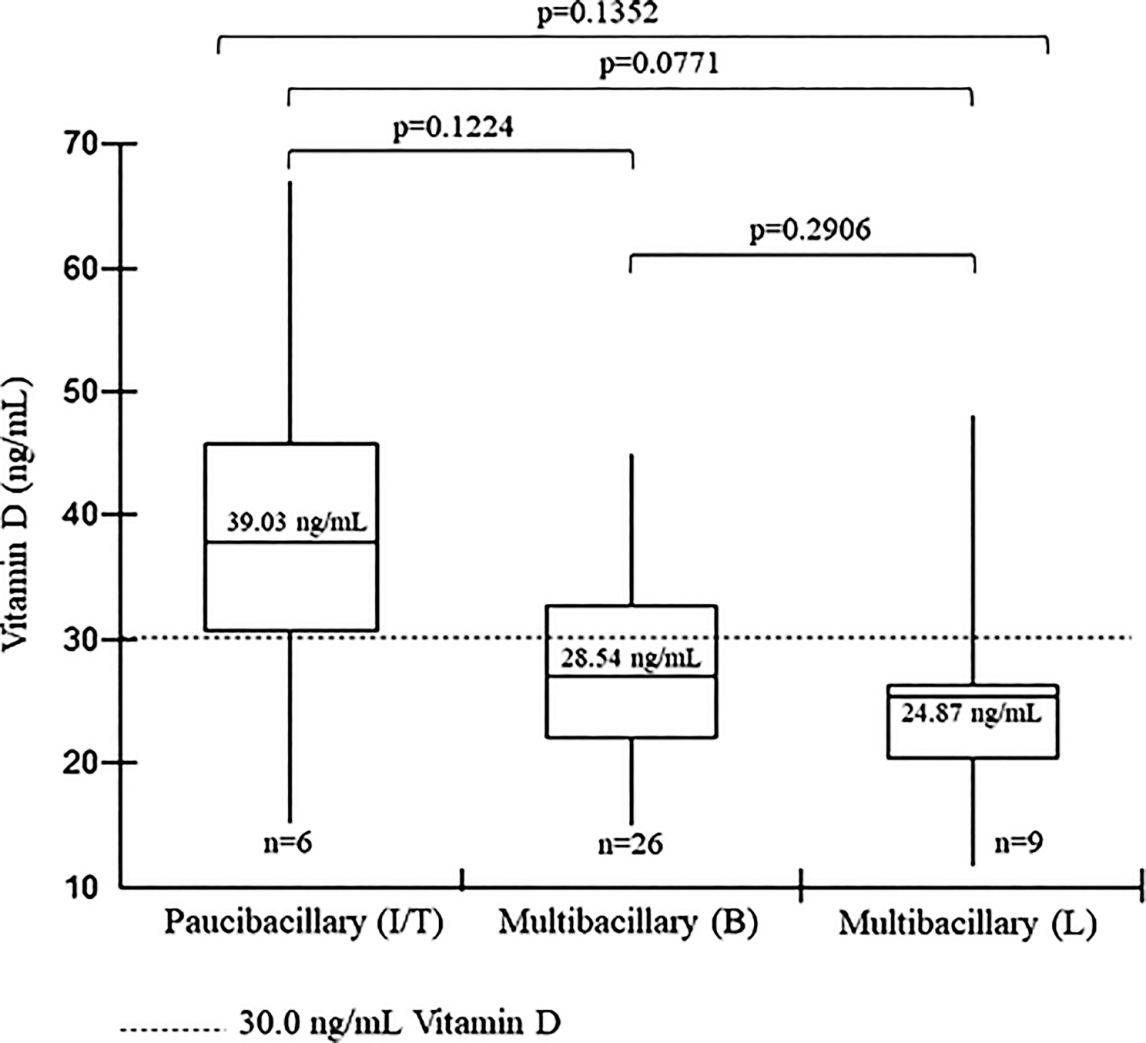
Vitamin D dosages and clinical form in patients diagnosed with leprosy treated in Marabá, Pará. Box Plot. Two-tailed Mann-Whitney test and Kruskal-Wallis test. I: indeterminate; T: tuberculoid; B: borderline; L: lepromatous

The measurement of 25-hydroxyvitamin D was carried out in all research participants and associated with clinical forms, leprosy reactions and positivity for autoantibodies. In the vitamin D dosage, we found that 25 (61.0%) participants had a dosage below 30.0 ng/mL, which characterizes vitamin D deficiency for the population at risk. The overall average vitamin D level was 29.3 ng/mL. When participants were divided according to clinical form, the mean level was 43.8 ng/mL in the tuberculoid form, 28.5 ng/mL in the borderline form, and 24.9 ng/mL in the lepromatous form. Only one participant was classified as indeterminate, with a vitamin D level of 15.0 ng/mL. In Fig. 1, this indeterminate case was grouped with the tuberculoid form, forming the paucibacillary group. In Group 1 (Paucibacillary – indeterminate/tuberculoid), vitamin D levels ranged from 15.0 to 67.0 ng/mL. In Group 2 (Borderline – multibacillary), levels ranged from 14.6 to 44.9 ng/mL, and in Group 3 (Lepromatous – multibacillary), from 11.5 to 47.9 ng/mL. The Fig. 1 presents the median measurements and quartiles of vitamin D dosages in the participants and the Table 4 the data that originated this figure.

**Table 4:** Vitamin D dosage and clinical form in patients diagnosed with leprosy treated in Marabá

Group	Mean (M) ± SD
Paucibacillary (I/T)	39.03 ± 17.6
Multibacillary (B)	28.54 ± 8.9
Multibacillary (L)	24.87 ± 10.5

SD: Standard Deviation

When comparing vitamin D dosage and clinical forms, we found no statistically significant difference between groups 1 and 2 (*p* = 0.1224), 1 and 3 (*p* = 0.0771), or 2 and 3 (*p* = 0.2906) using the Mann-Whitney test, two-tailed analysis. We also found no difference in the combination of all clinical forms (*p* = 0.1352) using the Kruskal-Wallis test.

## Discussion

This study included 41 individuals diagnosed with leprosy at a Family Health Public Unit in Marabá. Most participants were over 15 years old, but 19.5% were younger, a higher percentage than reported in other studies (7.8% and 6.9%) [32, 33]. Additionally, 87.5% of minors in our sample received multibacillary therapy, contrasting with findings in which most patients under 15 were paucibacillary [32, 34].

The detection rates in children under 15 years of age, as well as the high number of multibacillary patients, demonstrate the existence of high endemicity of leprosy in this region. Studies demonstrate that the maintenance of the disease has always been linked to the migration process of people between geographic territories, due to economic activities, such as mining and farming, which cause disorderly population growth in peripheral areas of medium and large municipalities [35, 36].

According to Silva [37], Marabá, in southeastern Pará, experienced intense economic cycles and presents spatial accessibility due to roads and rivers, which facilitated population influx. However, this rapid growth was not followed by adequate investments in housing and living conditions [37, 38]. The geographic pattern of occupation may contribute to the persistence of risk factors for leprosy transmission [35, 38]. Studies associate detection rates with living conditions and land occupation [36]. Consequently, Brazil’s highest concentration of cases is in the Legal Amazon, especially in southeastern and central-southern Pará [38].

The values presented in this study show a high rate of infection in children, as they were exposed to the pathogen very early. Barreto and collaborators [39] emphasized that there is a high rate of subclinical, undiagnosed leprosy in children in the Amazon. This may justify the large number of multibacillary children under 15 years of age in our study, since clinical manifestations are greater in these patients.

The predominance of multibacillary cases in this study is consistent with findings from other regions of Brazil [40–42]. We observed that 82.9% of participants needed to leave their neighborhood to access care, suggesting barriers to early diagnosis and treatment. Furthermore, 39.0% of cases took more than 24 months to receive diagnosis, which may contribute to greater transmission. Similar limitations in access and diagnostic capacity were reported in Manaus [43]. Vieira et al. [34] emphasized the importance of active case finding and contact examination, yet in our study only 29.3% underwent contact evaluation and no cases were detected through active search, indicating weaknesses in local control efforts.

Neglected diseases are closely linked to poverty, and in this study we found significant associations with low education (41.5%, *p* = 0.0025), low income (46.3%, *p* = 0.0089), precarious housing (46.3%, *p* = 0.0002), lack of piped water with use of wide-mouth wells (80.5%, *p* < 0.0001), and absence of sewage systems. These findings reinforce previous evidence connecting unfavorable socioeconomic conditions to leprosy incidence [44, 45]. Nery et al. [45] reported that individuals in poverty may have up to eight times greater risk of infection.

Among participants, 73.2% reported contact with infected individuals, and most had at least one BCG scar. Although our data did not show a protective effect, recent studies suggest that BCG may reduce disease manifestation in chronically exposed individuals, especially younger patients [46–49]. Beyond tuberculosis prevention, BCG may contribute to immunoprophylaxis against leprosy, though evidence remains under evaluation [50].

Spontaneous demand was the predominant mode of detection in this study (87.8%, *p* < 0.0001), aligning with national data showing it as the main entry form in the northern region between 2017 and 2021 [51]. This suggests that the population recognizes basic signs of leprosy, but reinforces the need for expanded health education and studies on maintaining passive surveillance, as indicated by the Ministry of Health [51].

We found a prevalence of 21.9% of autoantibodies in the total number of research participants. The autoantibodies investigated in our study were anti-cell antibodies (ANA HEp-2), anti-cardiolipin IgG antibodies (aCL), anti-β2-glycoprotein-I IgM antibodies (anti-β2-GPI IgM), anti-β2-glycoprotein-I IgG antibodies (anti-β2-GPI IgG) and rheumatoid factor (RF). There were positive results for all the autoantibodies mentioned, but anti-β2-GPI IgM and ANA (AC-2, AC-4 and AC-20) stand out, both in 14.6% of participants.

Rheumatoid factor (RF) and anti-CCP are markers used in rheumatoid arthritis diagnosis, but may also appear in leprosy. In our study, only one participant (2.4%) showed RF > 14 IU/mL, and this patient was experiencing type 2 reaction with joint symptoms. Dionello et al. [52] reported higher prevalences of RF (41.2%) and anti-CCP (9.3%) without association to reactional states. Other studies indicate frequent RF positivity but negative anti-CCP in leprosy, highlighting the value of evaluating both markers in inconclusive cases with joint involvement [53].

In this study, antiphospholipid autoantibodies were detected at low frequencies: aCL IgG (2.4%), anti-β2-GPI IgM (14.6%) and anti-β2-GPI IgG (4.9%), similar to other findings showing higher prevalence of anti-β2-GPI in leprosy [54–56]. All patients in type 2 reaction were positive for anti-β2-GPI IgM, though sample size limited statistical testing. Among cured individuals, anti-β2-GPI IgM persisted in 18.2%, consistent with studies indicating that antiphospholipid antibodies may not be transient in leprosy [54, 57, 58]. Evidence also shows IgM is more frequent than IgG in these patients [59], explaining the low aCL levels. Unlike APS, thrombotic events are not typical in leprosy despite antibody positivity, the presence of IgM is suggesting a T-cell-independent immune response to tissue damage [55, 57].

The presence of anti-β2-GPI IgM antibodies was statistically significant in patients with lepromatous leprosy, likely related to their high bacillary burden [57]. Although thrombotic events are uncommon in leprosy, Silva et al. [60] observed that multibacillary patients may develop a pro-coagulant profile associated with antiphospholipid antibodies and increased fibrinogen, von Willebrand factor, and tissue factor. A rare case has been reported in which a multibacillary patient—positive for anti-β2-GPI IgM and other autoantibodies—developed deep vein thrombosis and was diagnosed with secondary antiphospholipid syndrome (APS) [61].

Indirect immunofluorescence on HEp-2 cells was used to detect anti-cell autoantibodies (ANA), allowing visualization of nuclear and cytoplasmic reactivity. Six participants (14.6%) tested positive, with three fluorescence patterns identified: nuclear dense fine speckled (AC-2), nuclear fine speckled (AC-4), and mixed AC-4 with cytoplasmic fine speckled (AC-20). Titers and prevalences were 1/320 (2.4%), 1/80 (9.8%), and 1/320 (2.4%), respectively.

This ANA prevalence was lower than that reported by Bichara et al. (33.3%) [15], which may relate to methodological differences, as they used 1/40 serum dilution, while we adopted 1/80 dilution [62]. This approach better reflects clinical practice in differential diagnosis of autoimmune diseases but may reduce autoantibody detection. A study using the same 1/80 cutoff reported 2.0% positivity [16]. Ribeiro et al. [63] found 25.3% ANA positivity in leprosy patients, although dilution titer was not specified.

The AC-2 nuclear pattern, presented by anti-DFS70 autoantibodies, appears in several clinical situations, symptomatic or not, being the main pattern of immunofluorescence in healthy patients, without clinical manifestations or any complaints of autoimmune disease [64, 65]. These autoantibodies bind to the 70 kDa DSF protein, which resembles the p75 transcription factor, called LEDGF/p75 [65]. This factor has been the target of studies related to the human immunodeficiency virus (HIV), as it is involved in the viral DNA integration process [66].

In this study, the AC-2 pattern occurred in one female participant with a borderline form of leprosy before treatment. High ANA titers like this can appear in individuals without autoimmune disease symptoms [64]. Evidence shows that the AC-2 pattern in leprosy is not associated with the DSF70 antigen [67]. Studies recommend simultaneous ANA and anti-DSF70 testing, as anti-DSF70 positivity may help exclude autoimmune disorders [65, 67, 68]. A retrospective analysis found that only 24% of AC-2–positive patients developed autoimmune disease after two years [69]. Thus, in clinical practice, this finding should be complemented by anti-DSF70 testing and symptom evaluation, particularly given the patient’s joint complaints.

Cytoplasmic fluorescence patterns are frequently observed in leprosy, as described by Bichara et al. [15]. Although some studies report ANA positivity—such as Ribeiro et al. [14], who identified the AC-4 pattern in six patients—current literature is still insufficient to establish a clear association between nuclear or cytoplasmic autoantibodies and rheumatic manifestations or leprosy pathogenesis. It is worth noting that all participants who tested positive for ANA presented with the multibacillary, borderline, and lepromatous forms, with a higher frequency observed in patients with the lepromatous form (*n* = 4, 44.4% *p* = 0.0148). ANA positivity in leprosy is frequently reported in isolated case studies, often contributing to diagnostic confusion and delayed treatment—an issue that may lead to irreversible dermatoneurological damage [70, 71]. Clinical presentations can overlap with systemic lupus erythematosus, including arthritis, photosensitivity, malar rash, and positivity for ANA, antiphospholipid antibodies, and rheumatoid factor [9]. Therefore, autoantibody-positive patients should be carefully evaluated.

The anti-inflammatory role of vitamin D in immune regulation has been widely investigated, and its supplementation has been applied to various infectious diseases [21]. In our study, 61.0% (*n* = 25) of participants showed vitamin D deficiency (< 30.0 ng/mL), with a mean level of 29.3 ng/mL, higher than the deficiency rate reported by Ribeiro et al. [63] (35.6%). As both studies were conducted in Amazonian regions, sun exposure does not explain this difference, and the lack of data on supplementation also limits comparison. These findings highlight the need for future research exploring possible genetic variability in vitamin D metabolism between populations.

In our study, 28.0% (*n* = 7) of participants with vitamin D deficiency were positive for autoantibodies, with ANA and anti-β2 GPI IgM being most frequent. Ribeiro et al. [63] suggest that vitamin D deficiency may promote autoantibody production due to its immunomodulatory effect on B cells. Vitamin D levels are inversely related to clinical activity in autoimmune diseases [72], and evidence indicates that it inhibits B-cell differentiation into plasma cells, reducing antibody production, including self-reactive ones [73]. Associating autoantibody findings with vitamin D levels, we observed that paucibacillary patients, who showed higher plasma vitamin D concentrations (mean: 39.0 ng/mL), presented no autoantibody expression. Although the small sample size limited statistical analysis, these results support future research exploring the relationship between vitamin D status, clinical forms, and autoimmunity in leprosy.

Regarding leprosy reactions, all participants in reactional states (type 1 and type 2) presented vitamin D levels below 30.0 ng/mL. This finding aligns with Mandal et al. [30], who also observed hypovitaminosis D in all reactional patients in India. The authors further reported reduced VDR expression in this group, suggesting that VDR levels may influence the complexity and severity of leprosy progression.

Our study has some limitations due to being a cross-sectional study. It is suggested that a randomized clinical trial be conducted to better evaluate the relationship between vitamin D, assessing sun exposure and supplementation in leprosy patients. Furthermore, because this study relies on secondary data, such as patient records from a health unit, we encountered a lack of essential data for evaluating the action of vitamin D on the bacteria, such as the bacillary index. In addition, a study with a larger sample size and a control group is suggested, as the small number of participants limited the application of statistical tests for inferences and associations.

## Conclusion

We conclude that leprosy endemicity in Marabá reflects characteristics of neglected diseases, marked by low education, reduced income and limited housing conditions, with multibacillary forms predominating. The high frequency of infection in individuals under 15 years highlights the need for future studies on subclinical leprosy in children from this region. Autoantibodies were detected in local patients, particularly ANA and anti-β2-GPI IgM, while paucibacillary individuals showed higher vitamin D levels and no autoantibody expression. In contrast, multibacillary participants, especially lepromatous, more frequently presented with vitamin D deficiency and autoantibodies.

The study also identified low vitamin D concentrations in reactional patients, reinforcing the need to explore mechanisms linking inflammation, autoimmunity and vitamin D in M. leprae infection. Although the sample size was small and limits broad statistical inference, the findings are relevant and original for this population, providing valuable evidence that may guide future research and support deeper investigations into immune response and clinical variation in leprosy.

## Data Availability

The datasets used and/or analysed during the current study are available from the corresponding author on reasonable request.

## Abbreviations

Anti: aCL Anti-cardiolipin
AC: 2 Nuclear dense fine speckled
AC: 4 Nuclear fine speckled
AC: 20 Mixed AC-4 and cytoplasmic fine speckled
ANA Anti: Cell antibodies
ANCA Anti: Neutrophil cytoplasmic antibodies
anti: β2GPI Anti-β2 glycoprotein I
Anti: CCP Anti-Cyclic Citrullinated Peptide
Anti: DSF70 Anti-Dense Fine Speckled 70 kDa
Anti: DSF70 Anti-Dense Fine Speckled 70 kDa
APS: Antiphospholipid syndrome
BCG vaccine: Bacille Calmette-Guérin vaccine
ENL: *Erythema Nodosum Lepromatosum*
HEp-2 cells: Human Epithelial type 2 cells
HIV: Human immunodeficiency vírus
*M. leprae*: *Mycobacterium leprae*
NK: Natural Killer Cells
R1: Type 1 reaction or reverse reaction
R2: Type 2 reaction
RF: Rheumatoid fator
SLE: Systemic lupus erythematosus
Th1: T helper 1 lymphocyte
Th2: T helper 2 lymphocyte
VD: Vitamin D
VD: Vitamin D receptor

## References

[1] Froes LAR, Sotto MN, Trindade MAB. Leprosy: clinical and immunopathological characteristics. Bras Dermatol. 2022;97:338. 10.1016/J.ABD.2021.08.006. 10.1016/j.abd.2021.08.006 PMC9133310 35379512

[2] Cabral N, de Figueiredo V, Gandini M, de Souza CF, Medeiros RA, Lery LMS, et al. Modulation of the response to Mycobacterium leprae and pathogenesis of leprosy. Front Microbiol. 2022;13:918009. 10.3389/FMICB.2022.918009. 35722339 10.3389/fmicb.2022.918009 PMC9201476

[3] Teixeira CSS, Pescarini JM, Alves FJO, Nery JS, Sanchez MN, Teles C, et al. Incidence of and factors associated with leprosy among household contacts of patients with leprosy in Brazil. JAMA Dermatol. 2020;156:640–8. 10.1001/jamadermatol.2020.0653. 32293649 10.1001/jamadermatol.2020.0653 PMC7160739

[4] Bhandari JAMRB, Awais M, Robbins BA, Gupta V, Leprosy. StatPearls [Internet] 2022. Available at: https://www.ncbi.nlm.nih.gov/books/NBK559307/. 32644733

[5] Mi Z, Liu H, Zhang F. Advances in the immunology and genetics of leprosy. Front Immunol. 2020;11:1–15. 10.3389/fimmu.2020.00567. 32373110 10.3389/fimmu.2020.00567 PMC7176874

[6] Sadhu S, Mitra DK. Emerging concepts of adaptive immunity in leprosy. Front Immunol. 2018;9. 10.3389/fimmu.2018.00604. 10.3389/fimmu.2018.00604 PMC5900054 29686668

[7] da Brasil M. S (BR). Guide to leprosy control. Leprosy Control; 2002.

[8] Maymone MBC, Venkatesh S, Laughter M, Abdat R, Hugh J, Dacso MM, et al. Leprosy: treatment and management of complications. J Am Acad Dermatol. 2020;83:17–30. 10.1016/j.jaad.2019.10.138. 32244016 10.1016/j.jaad.2019.10.138

[9] El- Gendy H, El- Gohary RM, Shohdy KS, Ragab G. Leprosy masquerading as systemic rheumatic diseases. JCR: J Clin Rheumatology. 2016;22:264–71. 10.1097/RHU.0000000000000379. 10.1097/RHU.0000000000000379 27464771

[10] Naafs B, van Hees CLM. Leprosy type 1 reaction (formerly reversal reaction). Clin Dermatol. 2016;34:37–50. 10.1016/j.clindermatol.2015.10.006. 26773622 10.1016/j.clindermatol.2015.10.006

[11] Talhari C, Talhari S, Penna GO. Clinical aspects of leprosy. Clin Dermatol. 2015;33:26–37. 10.1016/j.clindermatol.2014.07.002. 25432808 10.1016/j.clindermatol.2014.07.002

[12] Pawar M, Zawar V. Mid -borderline leprosy masquerading as an overlap syndrome. Rheumatology. 2018;57:1686–8. 10.1093/rheumatology/key125. 29718382 10.1093/rheumatology/key125

[13] Zhang G, Jin H, Chen H, Lu Q. Cutaneous nodules with positive autoantibodies: histoid leprosy. Lancet. 2015;386:1915–6. 10.1016/S0140-67361500678-9. 10.1016/S0140-6736(15)00678-9 26843313

[14] Ribeiro SLE, Pereira HLA, Silva NP, Sato EI. Autoantibodies in patients with leprosy, with and without joint involvement, in the state of Amazonas. Rev Bras Rheumatol. 2009;49:547–61. http://www.scielo.br/pdf/rbr/v49n5/v49n5a06.pdf.

[15] Bichara CNC, Bichara CDA, Tostes C, Povoa MM, Quaresma JAS, Xavier MB. Prevalence of autoantibodies against cellular antigens in patients with HIV and leprosy coinfection in the Amazon region multilingual abstracts. Infect Dis Poverty. 2017;6:80. 10.1186/s40249-017-0294-2. 28566085 10.1186/s40249-017-0294-2 PMC5452639

[16] Neder L, Rondon DA, Cury SS, Da Silva CA. Musculoskeletal manifestations and autoantibodies in children and teenagers with leprosy. J Pediatr (Rio J). 2014;90:457–63. 10.1016/j.jped.2014.01.007. 24709568 10.1016/j.jped.2014.01.007

[17] Cusick MF, Libbey JE, Fujinami RS. Molecular mimicry as a mechanism of autoimmune disease. Clin Rev Allergy Immunol. 2012;42:102–11. 10.1007/s12016-011-8294-7. 22095454 10.1007/s12016-011-8294-7 PMC3266166

[18] Ao T, Kikuta J, Ishii M. The effects of vitamin D on immune system and inflammatory diseases. Biomolecules. 2021;11. 10.3390/biom11111624. 10.3390/biom11111624 PMC8615708 34827621

[19] White JH. Emerging roles of vitamin D- induced antimicrobial peptides in antiviral innate immunity. Nutrients. 2022;14. 10.3390/nu14020284. 10.3390/nu14020284 PMC8779757 35057465

[20] Das LM, Binko AM, Traylor ZP, Peng H, Lu KQ. Vitamin D improves sunburns by increasing autophagy in M2 macrophages. Autophagy. 2019;15:813–26. 10.1080/15548627.2019.1569298. 30661440 10.1080/15548627.2019.1569298 PMC6526871

[21] Aranow C. Vitamin D and the immune system. J Investig Med. 2011;59:881–6. 10.231/JIM.0b013e31821b8755. 21527855 PMC3166406

[22] Neela VSK, Suryadevara NC, Shinde VG, Pydi SS, Jain S, Jonnalagada S, et al. Association of Taq I, Fok I and apa I polymorphisms in vitamin D receptor (VDR) gene with leprosy. Hum Immunol. 2015;76:402–5. 10.1016/j.humimm.2015.04.002. 25890006 10.1016/j.humimm.2015.04.002

[23] Singh I, Lavania M, Pathak VK, Ahuja M, Turankar RP, Singh V, et al. VDR polymorphism, gene expression and vitamin D levels in leprosy patients from North Indian population. PLoS Negl Trop Dis. 2018;12:e0006823. 10.1371/journal.pntd.0006823. 30481178 10.1371/journal.pntd.0006823 PMC6286024

[24] Araújo TG, Oliveira GP, de Matos Oliveira F, Neves AF, Soares Mota ST, Goulart IMB, et al. A novel vitamin D receptor polymorphism associated with leprosy. J Dermatol Sci. 2018;89:304–7. 10.1016/j.jdermsci.2017.12.007. 29290530 10.1016/j.jdermsci.2017.12.007

[25] Pinto Paz JL, do Perpétuo Socorro Corrêa Amador Silvestre M, Moura LS, Furlaneto IP, Rodrigues YC, Batista Lima KV, et al. Association of the polymorphism of the vitamin D receptor gene (VDR) with the risk of leprosy in the Brazilian Amazon. Biosci Rep. 2021;41:1–9. 10.1042/BSR20204102. 10.1042/BSR20204102 PMC8264180 34143211

[26] Pepineli AC, Alves HV, Tiyo BT, Macedo LC, Visentainer L, de Lima Neto QA, et al. Vitamin D receptor gene polymorphisms are associated with leprosy in Southern Brazil. Front Immunol. 2019;10. 10.3389/fimmu.2019.02157. 10.3389/fimmu.2019.02157 PMC6787522 31636627

[27] Luong KVQ, Nguyen LTH. Role of the vitamin D in leprosy. American journal of themedical. Sciences. 2012;343:471–82. 10.1097/MAJ.0b013e318232a6cf. 10.1097/MAJ.0b013e318232a6cf 22170299

[28] Grossi de Oliveira AL, Chaves AT, Cardoso MS, Pinheiro GRG, Antunes DE, de Grossi MA. Reduced vitamin D receptor (VDR) and Cathelicidin antimicrobial peptide (CAMP) gene expression contribute i’m the maintenance of inflammatory immune response in leprosy patients. Microbes Infect. 2022;24:104981. 10.1016/j.micinf.2022.104981. 35462022 10.1016/j.micinf.2022.104981

[29] Grossi de Oliveira AL, Chaves AT, Santos Cardoso M, Gomide Pinheiro GR, Parreiras de Jesus AC, de Faria Grossi MA, et al. Hypovitaminosis D and reduced Cathelicidin are strongly correlated during the multidrug therapy against leprosy. Microb Pathog. 2020;147:104373. 10.1016/j.micpath.2020.104373. 32645421 10.1016/j.micpath.2020.104373

[30] Mandal D, Reja AHH, Biswas N, Bhattacharyya P, Patra PK, Bhattacharya B. Vitamin D receptor expression levels determine the severity and complexity of illness progression among leprosy reaction patients. New Microbes New Infect. 2015;6:35–9. 10.1016/j.nmni.2015.04.001. 26106480 10.1016/j.nmni.2015.04.001 PMC4475695

[31] Pinheiro BV. da S. Spatial distribution of leprosy and its relationship with socioeconomic variables and public policies, in three municipalities in the state of Pará. Dissertation (Master’s Degree in Health, Environment and Society in the Amazon) - Institute of Health Sciences, University Federal Do Pará 2017.

[32] Neto PML, da Silva AR, Santos LH, Dos, Lima RJCP, Tauil PL. Gonçalves E Da G do R. Leprosy in children under 15 years of age in a municipality in Northeastern brazil: evolutionary aspects from 2003 to 2015. Rev Social Bras Med Trop. 2020;53:1–5. 10.1590/0037-8682-0515-2020. 10.1590/0037-8682-0515-2020 PMC7723375 33263688

[33] Lana FCF, Amaral EP, Lanza FM, Lima PL, Carvalho ACN, Diniz LG. Leprosy in children under 15 years of age in Vale do Jequitinhonha, Minas Gerais, Brazil. Rev Bras Enferm. 2007;60:696–700. 10.1590/S0034-71672007000600014. 18472544 10.1590/s0034-71672007000600014

[34] Vieira MCA, Nery JS, Paixão ES, Freitas de Andrade KV, Oliveira Penna G, Teixeira MG. Leprosy in children under 15 years of age in brazil: A systematic review of the literature. PLoS Negl Trop Dis. 2018;12:1–13. 10.1371/journal.pntd.0006788. 10.1371/journal.pntd.0006788 PMC6168122 30278054

[35] Pereira WMM, Braga RL, da Silva ER, dos Santos JNG, Vinente Neto BF, Mota JVF, et al. Leprosy and migration: Spatial correlation in a hyperendemic state in the Brazilian Amazon. Res Soc Dev. 2021;10:e1810111164. 10.33448/rsd-v10i1.11164.

[36] Silva RDX, Ignotti E, Souza-Santos R, de Hacon S. S. Leprosy, social conditions and deforestation in the Brazilian Amazon. Rev Panam Salud Publica. 2010;27:268 – 75. Available at: http://www.scielosp.org/scielo.php?script=sci_arttext&pid=S1020-49892010000400005. 10.1590/s1020-49892010000400005 20512229

[37] de Silva S. Production of urban space in Marabá (PA): trajectories and processes. Geopauta. 2022;6:e10094. 10.22481/rg.v6.e2022.e10094.

[38] Rodrigues RN, Leano HA, de Bueno M, de C I, Araújo KM da, Lana FA. FCF. High- risk areas of leprosy in Brazil between 2001–2015. *Rev Bras Enferm* 2020;73. 10.1590/0034-7167-2018-0583 10.1590/0034-7167-2018-0583 32294707

[39] Barreto JG, Guimarães L, de Frade S, Rosa MAC, Salgado PS. High rates of undiagnosed leprosy and subclinical infection amongst school children in the Amazon region. Mem Inst Oswaldo Cruz. 2012;107:60–7. 10.1590/S0074-02762012000900011. 23283455 10.1590/s0074-02762012000900011

[40] de Moraes PC, Eidt LM, Koehler A, Ransan LG, Scrofeneker ML. Epidemiological characteristics of leprosy from 2000 to 2019 in a state with low endemicity in Southern Brazil. Bras Dermatol. 2023. 10.1016/j.abd.2022.08.009. 10.1016/j.abd.2022.08.009 PMC10404493 37120406

[41] Machado LMG, dos Santos ES, Cavaliero A, Steinmann P, Ignotti E. Spatio -temporal analysis of leprosy risks in a municipality in the state of Mato Grosso- Brazilian amazon: results from the post- exposure leprosy prophylaxis program in Brazil. Infect Dis Poverty. 2022;11. 10.1186/s40249-022-00943-7. 10.1186/s40249-022-00943-7 PMC8862266 35193684

[42] Moura RS, Penna GO, Vaz Cardoso LP, De Andrade Pontes MA, Cruz R, De Sá Gonçalves H, et al. Description of leprosy classification at baseline among patients enrolled at the uniform multidrug therapy clinical trial for leprosy patients in Brazil. Am J Trop Med Hyg. 2015;92:1280–4. 10.4269/ajtmh.14-0049. 25940192 10.4269/ajtmh.14-0049 PMC4458838

[43] Silva DSe, Palheta Júnior JIL, Pedrosa VL, Talhari C. Leprosy in the state of amazonas: is there actually a decrease in its incidence and prevalence ? Bras Dermatol. 2022;97:513–5. 10.1016/j.abd.2021.01.007. 10.1016/j.abd.2021.01.007 PMC9263664 35672159

[44] Kerr-Pontes LRS, Barreto ML, Evangelista CMN, Rodrigues LC, Heukelbach J, Feldmeier H. Socioeconomic, environmental, and behavioral risk factors for leprosy in North- East Brazil: results of a case- control study. Int J Epidemiol. 2006;35:994–1000. 10.1093/ije/dyl072. 16645029 10.1093/ije/dyl072

[45] Nery JS, Ramond A, Pescarini JM, Alves A, Strina A, Ichihara MY, et al. Socioeconomic determinants of leprosy new case detection in the 100 million Brazilian cohort: a population-based linking study. Lancet Glob Health. 2019;7:e1226–36. 10.1016/S2214-109X(19)30260-8. 31331811 10.1016/S2214-109X(19)30260-8 PMC6688099

[46] van Hooij A, van den Eeden SJF, Khatun M, Soren S, Franken KLMC, Chandra Roy J, et al. BCG- induced immunity profiles in households contacts of leprosy patients differentiate between protection and disease. Vaccine. 2021;39:7230–7. 10.1016/j.vaccine.2021.10.027. 34688497 10.1016/j.vaccine.2021.10.027

[47] Araujo S, Rezende MMF, de Sousa DCR, Rosa MR, dos Santos DC, Goulart LR, et al. Risk- benefit assessment of Bacillus Calmette-Guérin vaccination, anti-phenolic glycolipid I serology, and Mitsuda test response: 10-year follow-up of household contacts of leprosy patients. Rev Social Bras Med Trop. 2015;48:739–45. 10.1590/0037-8682-0245-2015. 10.1590/0037-8682-0245-2015 26676499

[48] Gomes RR, Antunes DE, dos Santos DF, Sabino EFP, Oliveira DB, Goulart IMB. BCG vaccine and leprosy household contacts: protective effect and probability i’m becoming sick during follow-up. Vaccine. 2019;37:6510–7. 10.1016/j.vaccine.2019.08.067. 31500969 10.1016/j.vaccine.2019.08.067

[49] Cuevas NC, Cardenas VM. Bacillus of calmette and Guerin (BCG) and the risk of leprosy in Ciudad Del Este, Paraguay, 2016–2017. Epidemiol Health 2021;43. 10.4178/epih.e2021060. 10.4178/epih.e2021060 PMC8850947 34525502

[50] Yamazaki-Nakashimada MA, Unzueta A, Berenise Gámez -González L, González- Saldaña N, Sorensen RU. BCG: a vaccine with multiple faces. Hum Vaccine Immunother. 2020;16:1841–50. 10.1080/21645515.2019.1706930. 10.1080/21645515.2019.1706930 PMC7482865 31995448

[51] BRASIL M da S. Leprosy Epidemiological Bulletin 2023.

[52] Dionello CF, Rosa Utiyama SR, Radominski SC, Stahlke E, Stinghen ST, de Messias- Reason IJ. Evaluation of rheumatoid factor and anti-citrullinated peptide antibodies in relation i’m rheumatological manifestations in patients with leprosy from Southern Brazil. Int J Rheum Dis. 2016;19:1024–31. 10.1111/1756-185X.12668. 26250118 10.1111/1756-185X.12668

[53] Ribeiro SLE, Pereira HLA, Silva NP, Neves RMS, Sato EI. Anti-cyclic citrullinated peptide antibodies and rheumatoid factor in leprosy patients with articular involvement. Braz J Med Biol Res. 2008;41:1005–10. 10.1590/s0100-879x2008001100010. 19099154 10.1590/s0100-879x2008001100010

[54] Ribeiro SLE, Pereira HLA, Silva NP, Sato EI, Passos LFS, Dos-Santos MC. Long-term persistence of anti -β2 glycoprotein i in treated leprosy patients. Lupus. 2014;23:1249–51. 10.1177/0961203314529469. 25228717 10.1177/0961203314529469

[55] Ribeiro SLE, Pereira HLA, Silva NP, Souza AWS, Sato EI. Anti-b2-glycoprotein i antibodies are highly prevalent in a large number of Brazilian leprosy patients. Acta Reumatol Port. 2011;36:30–7. 21483278

[56] Loizou S, Singh S, Wypkema E, Asherson RA. Anticardiolipin, anti-b 2-glycoprotein I and antiprothrombin antibodies in black South African patients with infectious disease. Ann Rheum Dis. 2003;62:1106–11. 10.1136/ard.62.11.1106. 14583576 10.1136/ard.62.11.1106 PMC1754364

[57] Ribeiro SLE, Pereira HLA, Boechat AL, Silva NP, Sato EI, Das Graças Souza Cunha M, et al. Epidemiological, clinical and immune factors that influence the persistence of antiphospholipid antibodies in leprosy. Adv Rheumatol. 2019;59. 10.1186/s42358-019-0094-4. 10.1186/s42358-019-0094-4 31779703

[58] Yang X, Dong H, Kuang YQ, Yu XF, Long H, Zhang CY, et al. Long-term presence of autoantibodies in plasma of cured leprosy patients. Sci Rep. 2023;13. 10.1038/s41598-022-27256-x. 10.1038/s41598-022-27256-x PMC9816311 36604576

[59] Kelchtermans H, Pelkmans L, de Laat B, Devreese KM. IgG/ IgM antiphospholipid antibodies present in the classification criteria for the antiphospholipid syndrome: a critical review of their association with thrombosis. J Thromb Haemost. 2016;14:1530–48. 10.1111/jth.13379. 27279342 10.1111/jth.13379

[60] Silva DS da, Teixeira LAC, Beghini DG, Ferreira AT da S, Pinho M de, PS Rosa BM, et al. Blood coagulation abnormalities in multibacillary leprosy patients. PLoS Negl Trop Dis. 2018;12. 10.1371/journal.pntd.0006214. 10.1371/journal.pntd.0006214 PMC5863944 29565968

[61] Jones D, CA J, Joshi A, AS S, Kumar H. Deep vein thrombosis in lepromatous leprosy: a case report of secondary pediatric antiphospholipid syndrome. Cureus 2022. 10.7759/cureus.21361 10.7759/cureus.21361 PMC8853942 35198275

[62] Cruvinel W, de Andrade M, von Mühlen LEC, Dellavance CA, Ximenes A, Bichara AC. V Brazilian consensus guidelines for detection of anti-cell autoantibodies on hep-2 cells. Adv Rheumatol. 2019;59:28. 10.1186/s42358-019-0069-5. 31269997 10.1186/s42358-019-0069-5

[63] Ribeiro SLE, Pereira HLA, Mangueira CL, Ferreira CES, Rosseto E, Scheinberg M. The development of arthritis and antinuclear antibodies correlate with serum 25-hydroxyvitamin D levels in patients with leprosy. Ann Rheum Dis. 2012;71:2062–3. 10.1136/annrheumdis-2012-201485. 22730368 10.1136/annrheumdis-2012-201485

[64] Dellavance A, Leser PG, Andrade LEC. Importance of the fluorescence pattern in the interpretation of the FAN test - The case of the dense fine dotted pattern. Rev Assoc Med Bras. 2007;53:439–45. 10.1590/S0104-42302007000500021. 17952354 10.1590/s0104-42302007000500021

[65] Carter JB, Carter S, Saschenbrecker S, Goeckeritz BE. Recognition and relevance of Anti-DFS70 autoantibodies in routine antinuclear autoantibodies testing at a community hospital. Front Med (Lausanne). 2018;5. 10.3389/fmed.2018.00088. 10.3389/fmed.2018.00088 PMC5900435 29686987

[66] Passaes CPB, Cardoso CC, Caetano DG, Teixeira SLM, Guimarães ML, Campos DP, et al. Association of single nucleotide polymorphisms in the lens Epithelium-Derived growth factor (LEDGF/p75) with HIV-1 infection outcomes in Brazilian HIV-1 + Individuals. PLoS ONE. 2014;9:e101780. 10.1371/journal.pone. 25047784 10.1371/journal.pone.0101780 PMC4105638

[67] Ribeiro SLE, Nunes GPS, de Freitas HEG, Mangueira C, Scheinberg MA. Negative specificity for DSF70 in ANA positive leprosy will be. Clin Rheumatol. 2019;38:2953–5. 10.1007/s10067-019-04667-2. 31278511 10.1007/s10067-019-04667-2

[68] Conrad K, Röber N, Andrade LEC, Mahler M. The clinical relevance of Anti-DFS70 autoantibodies. Clin Rev Allergy Immunol. 2017;52:202–16. 10.1007/s12016-016-8564-5. 27350273 10.1007/s12016-016-8564-5

[69] Lundgren MC, Sapkota S, Peterson DJ, Crosson JT. The antinuclear antibody dense fine speckled pattern and possible clinical associations: an indication of a proinflammatory microenvironment. J Immunol Methods. 2021;488. 10.1016/j.jim.2020.112904. 10.1016/j.jim.2020.112904 33121975

[70] de Andrade TCPC, Vieira BC, Soares CT, Martins TY, Santiago TM, Barreto JA. Lepromatous leprosy simulation rheumatoid arthritis – Report of a neglected disease. Bras Dermatol. 2017;92:389–91. 10.1590/abd1806-4841.20175423. 10.1590/abd1806-4841.20175423 PMC5514583 29186255

[71] Gao W, Chen Z, Jiang H, Shi Y, Zhang W, Wang H. Diffuse multibacillary leprosy patient with lucio’s phenomenon and positive anticardiolipin antibody misdiagnosed as lupus erythematosus panniculitis in the people’s Republic of China. Infect Drug Resist. 2019;12:741–4. 10.2147/IDR.S202386. 31114260 10.2147/IDR.S202386 PMC6497491

[72] Athanassiou L, Kostoglou-Athanassiou I, Koutsilieris M, Shoenfeld Y. Vitamin D and autoimmune rheumatic diseases. Biomolecules. 2023;13:709. 10.3390/biom13040709. 37189455 10.3390/biom13040709 PMC10135889

[73] Yamamoto EA, Nguyen JK, Liu J, Keller E, Campbell N, Zhang CJ, et al. Low levels of vitamin D promotes memory B cells in lupus. Nutrients. 2020;12. 10.3390/nu12020291. 10.3390/nu12020291 PMC7070834 31978964

